# Reevaluating the diagnosis of right coronary artery absence: A thoughtful analysis of a case report

**DOI:** 10.1097/MD.0000000000043620

**Published:** 2025-08-08

**Authors:** Xiaowei Wang, Honggen Cui, Zhanwen Xu

**Affiliations:** a Department of Cardiology, Affiliated Hospital of Hebei University, Baoding, China.

**Keywords:** acute myocardial infarction, percutaneous coronary intervention, right coronary artery absence

## Abstract

**Rationale:**

This reevaluation challenges the diagnostic certainty of coronary angiography (CAG) and computed tomography angiography (CTA) in confirming right coronary artery (RCA) agenesis, as presented in Seok Oh et al’s case. It underscores the critical need for multimodal imaging to avoid misdiagnosis of rare coronary anomalies.

**Patient concerns:**

A 57-year-old male presented with severe chest pain lasting several hours, accompanied by hemodynamic instability and ST-segment elevation consistent with myocardial infarction.

**Diagnoses:**

CAG revealed total occlusion of the left circumflex artery (LCX) and suggested that the RCA territory was supplied by a superdominant LCX. Coronary CTA later indicated the absence of the RCA, though diagnostic uncertainties remained.

**Interventions:**

Emergent percutaneous coronary intervention was performed to address the LCX occlusion, resulting in clinical stabilization.

**Outcomes:**

Reanalysis of published CAG images revealed no definitive evidence of LCX extending to RCA territory. CTA suggested the “RCA-supplying branch” was likely a coronary vein, implying possible misinterpretation of a left-dominant system with anomalous small RCA.

**Lessons:**

This case highlights the challenges of diagnosing rare coronary anomalies and emphasizes the importance of multi-modal imaging for accurate evaluation and decision-making.

## 1. Introduction

Congenital agenesis of the right coronary artery is a rare coronary anomaly, with fewer than 80 cases reported in the literature. Typically, congenital agenesis of the right coronary artery is associated with a left-dominant coronary system, where the left coronary artery (LCA) supplies the myocardial territories traditionally served by the RCA. This anomaly can present with severe clinical outcomes, such as myocardial infarction, as demonstrated in the case by Seok Oh et al.^[1]^ The authors suggest that the patient’s myocardial infarction was caused by a total occlusion of the left circumflex artery (LCX), with a single coronary artery (SCA) anomaly due to the absence of the right coronary artery (RCA), and the RCA territory being supplied by a superdominant LCX. However, upon reviewing the coronary angiography (CAG) and coronary computed tomography angiography (CTA), we identified several concerns about the evidence presented and the interpretation of RCA absence.

## 2. Case report

The patient, a 57-year-old male, presented with a few hours of chest pain and was diagnosed with ST-elevation myocardial infarction. The initial diagnosis led to an emergent CAG, which revealed a total occlusion of the LCX. The authors report that the RCA was absent from the aortic root, and the LCX was supplying the RCA territory. The coronary CTA confirmed the absence of the RCA. The authors describe an absence of the RCA based on coronary CTA, with a large branch originating from the LCX that supplies the RCA territory.

**Figure 1.**
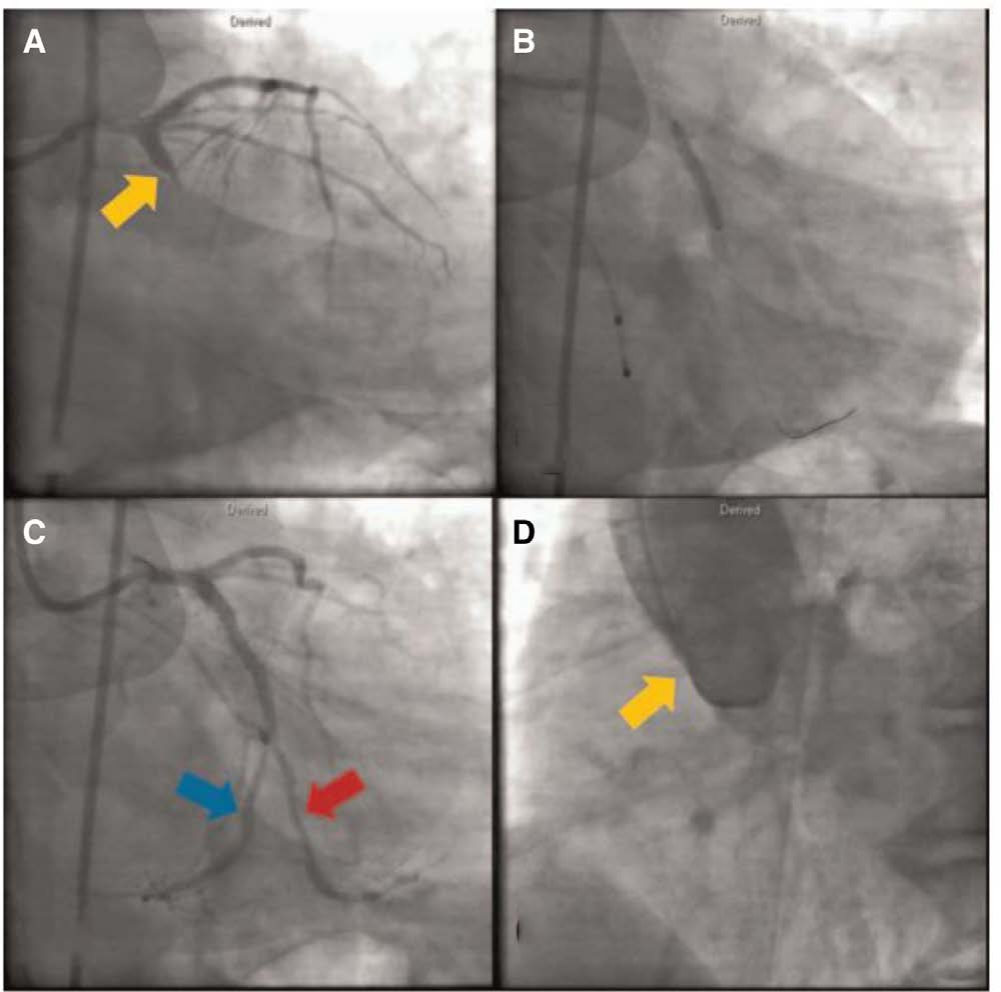
The main branch of the left circumflex artery (blue arrow, C) is shown terminating at the left ventricular lateral wall and does not extend into the right coronary artery distribution area. (A) Coronary angiogram showing total proximal occlusion of the left circumflex artery. (B) Postintervention angiogram demonstrating successful recanalization of the left circumflex artery. (D) Nonselective injection at the base of the aortic sinus; note the absence of a right coronary artery arising from the right coronary sinus. LCX = left circumflex artery, RCA = right coronary artery. Reused from Oh et al.^[1]^

**Figure 2.**
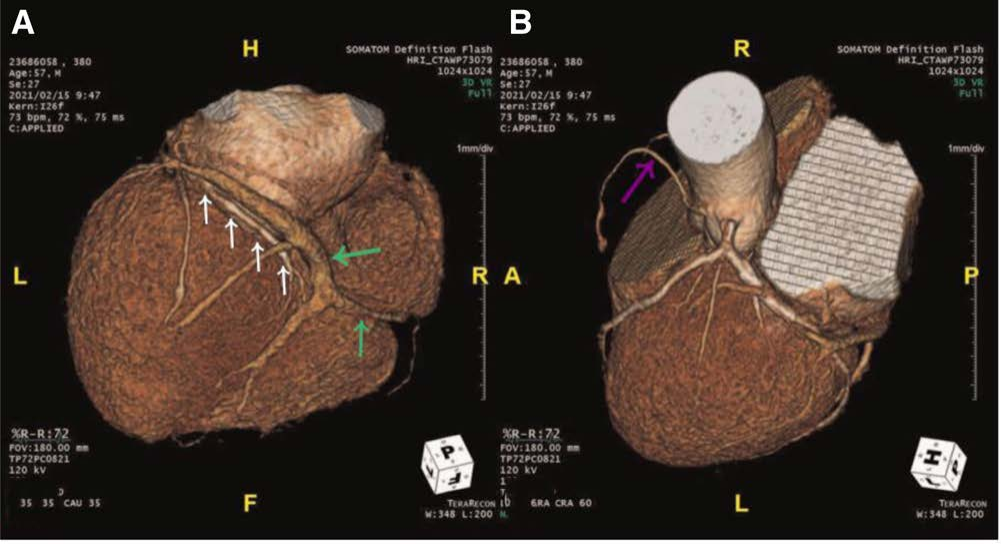
The white arrow indicates the course of the circumflex artery (LCX), which runs to the left atrioventricular groove and terminates at the posterior interventricular groove. The green arrow (A) points to the coronary vein, and the purple arrow (B) points to the right coronary artery (RCA). LCX = left circumflex artery, RCA = right coronary artery. Reused from Oh et al.^[1]^

Upon reviewing the angiographic images, we believe that the provided CAG images do not definitively demonstrate the absence of the RCA. In Figure 1, the original authors pointed out that the LCX has 2 large branches, 1 (red arrow) follows the LCX’s typical distribution path, while the other (blue arrow) follows the RCA’s distribution. However, the red arrow actually points to a branch of the obtuse marginal artery of the LCX, and the blue arrow refers to the main branch of the LCX. The main branch of the LCX (blue arrow) is shown terminating at the left ventricular lateral wall and does not extend into the RCA distribution area. The CAG image does not show any continuation into the RCA region, raising questions about the reported absence of the RCA. Furthermore, in the coronary CTA (Fig. 2), while the authors suggest that a large branch from the LCX is supplying the RCA territory, the precise identification of this branch is unclear. The CTA image shows the LCX (white arrow) running along the left atrioventricular groove, with no clear branch extending to the right atrioventricular groove, which is typically the area supplied by the RCA. Additionally, the vessel appearing in the right atrioventricular groove is likely a coronary vein (green arrow), not an artery, as it lacks the continuity with the LCX typical of an arterial vessel. The purple arrow we have marked indicates the RCA. Initial CAG failed to delineate the RCA ostium within the right coronary sinus, despite utilizing nonselective contrast injections via a pigtail catheter and systematic aortic root imaging in orthogonal projections. Subsequent coronary CTA, however, revealed a hypoplastic RCA with anomalous origin from the left coronary sinus.

## 3. Discussion

SCA is a rare coronary anomaly, with an incidence rate of 0.024% to 0.09% in patients undergoing CAG.^[2,3]^ The majority of SCA anomalies do not coexist with other cardiovascular malformations; however, a few cases have been reported in combination with conditions such as bicuspid aortic valve anomalies, transposition of the great arteries, and ventricular septal defects.^[3–5]^ The probability of a SCA originating from the right coronary sinus or left coronary sinus is similar. In patients with a single RCA due to the absence of the LCA, the LCA typically originates from the proximal part of the RCA, running between the aorta and the pulmonary artery conus, reaching the left side, and then branching into the left anterior descending artery and the left circumflex artery.^[6,7]^

In contrast, in patients with a single LCA due to the absence of the RCA, the RCA may be compensated by one of 3 mechanisms from the left coronary system:

The first mechanism involves the LCA originating proximally and then running toward the right side, reaching the usual RCA position and branching into the RCA’s various branches. This type is the most common, with the RCA typically arising from the proximal left anterior descending artery, followed by the left main coronary artery.^[2,8]^

The second mechanism involves the large distal LCX continuing retrogradely to give rise to the RCA branches. Seok Oh et al considered the patient’s SCA anomaly to be the second type. However, as we have analyzed above, the CAG and coronary CTA did not show the LCX extending into the RCA. Therefore, with a careful and thorough approach, we present this case for further analysis.^[9,10]^

The third mechanism is less common and involves the distal left anterior descending artery continuing, giving rise to the coronary branches that replace the RCA, such as the conus branch, right ventricular branch, posterior descending artery, and the left ventricular posterior branch, after passing through the mid and distal portions and around the apex of the heart.

CAG provides 2-dimensional images and cannot display the coronary artery’s origin, full course, termination points, and its spatial relationship with the heart’s great arteries in 3 dimensions. In contrast, coronary CTA offers indirect imaging, allowing the entire heart and coronary arteries to be visualized simultaneously. This method overcomes imaging and diagnostic challenges posed by variations in coronary artery origin and course, resulting in higher imaging success rates and diagnostic accuracy. By incorporating various post-processing techniques, coronary CTA enables physicians to perform a comprehensive and accurate analysis of SCA anomalies. Among these techniques, volume rendering (VR) imaging is the most important, intuitive, reliable, and commonly used for diagnosing SCA. VR imaging allows for clear visualization of the anomalous origin of the coronary artery, its full course within the epicardial fat space, its termination, and its spatial relationship with the heart’s great arteries. Maximum intensity projection images are useful in reflecting vascular stenosis, dilation, calcified plaques on vessel walls, and contrast agent filling in the lumen. Curved planar reformation can display the entire length of curved vessels in a single plane, aiding in the diagnosis of patients with concomitant coronary artery disease. VR is primarily used to observe the coronary artery’s opening and lumen from within. Additionally, coronary CTA can detect other related cardiac malformations or conditions. However, in this case, the CTA image shows a vessel that appears to be a coronary vein rather than an artery. Thus, we propose that the patient may have a left-dominant coronary system with a small anomalous RCA.

## 4. Conclusion

This case presents a unique presentation of a possible congenital anomaly of the coronary arteries. However, the imaging evidence provided does not convincingly demonstrate the complete absence of the RCA. Instead, we propose that the patient may have a left-dominant coronary system with a small anomalous RCA. Given the complexity of coronary anomalies, further imaging modalities and a more detailed analysis of the coronary anatomy are required to clarify the diagnosis. This case highlights the importance of precise imaging techniques in diagnosing rare coronary anomalies.

## 4.1. Limitations

Our analysis relies solely on published CAG/CTA images from Seok Oh et al Without access to raw imaging data or additional modalities, the possibility of a hypoplastic RCA cannot be definitively excluded. This constraint underscores the need for cautious interpretation of published images in complex anomalies.

## Author contributions

**Conceptualization:** Zhanwen Xu.

**Data curation:** Zhanwen Xu.

**Formal analysis:** Zhanwen Xu.

**Writing – original draft:** Xiaowei Wang, Honggen Cui, Zhanwen Xu.

**Writing – review & editing:** Xiaowei Wang, Honggen Cui, Zhanwen Xu.
